# Urokinase in the treatment of tuberculous pleurisy: a systematic review and meta-analysis

**DOI:** 10.1186/s12879-024-08975-0

**Published:** 2024-02-24

**Authors:** Wenyao Jing, Ruolan Weng, Ping Lin, Miao Luo

**Affiliations:** Affiliated Hospital of Guilin Medical University, Guilin, China

**Keywords:** Pleural tuberculosis, Urokinase, Randomized controlled trial, Meta

## Abstract

**Objective:**

To evaluate the efficacy of urokinase (UK) treatment for tuberculous pleural effusion (TPE).

**Methods:**

We searched Chinese biomedical literature database, WanFang data, CNKI, PubMed, EMbase, Web of Science and The Cochrane Library for the randomized controlled trials (RCTs) of urokinase treatment for tuberculous pleurisy from January 2000 to February 2023. Pleural tuberculosis, urokinase and randomized controlled trial were used as keywords. The eligible studies were meta-analyzed by using Revman 5.4.1: risk of bias was assessed, mean difference (MD) and 95% CI were used for continuous variables, pooled studies were conducted using random-effects or fixed-effects models, forest plots were drawn to analyze efficacy, and funnel plots were drawn to discuss publication bias.

**Results:**

Twenty-nine RCTs were included. The meta-analyzed results showed that, on the basis of routine anti-tuberculosis, comparison between the treatment group treated with urokinase and the control group treated with antituberculosis alone, the time of pleural effusion absorption [MD-5.82, 95%CI (− 7.77, − 3.87); *P*<0.00001] and the residual pleural thickness [MD-1.31, 95%CI (− 1.70, − 0.91); P<0.00001], pleural effusion drainage volume [MD 822.81, 95%CI (666.46,977.96); *P*<0.00001], FVC%pred [MD 7.95, 95%CI (4.51,11.40); *P*<0.00001], FEV1%pred [MD 12.67, 95%CI (10.09,15.24); *P*<0.00001] were significantly different.

**Conclusion:**

The clinical effect of urokinase is better than that of antituberculous therapy alone: it can increase total pleural effusion, decrease residual pleural thickness, improve the pulmonary function, and shorten the time of pleural effusion absorption.

**Supplementary Information:**

The online version contains supplementary material available at 10.1186/s12879-024-08975-0.

## Introduction

Tuberculous pleural effusion is the most common infectious pleural disease and one of the major respiratory diseases in China [1]. The global tuberculosis report 2022 shows that, an estimated 10.6 million people became ill with tuberculosis in 2021, and 1.6 million people died from tuberculosis in 2021, among which about 64,000 died in China [2]. Tuberculous pleurisy is more prevalent in those countries with high prevalence of tuberculosis, and in China, tuberculous pleurisy accounts for about 50% of pleural effusion cases [3]. The traditional treatment for TPE is systemic anti-tuberculosis therapy combined with local fluid extraction, but many patients may easily develop pleural hypertrophy, adhesions, and encapsulated effusion due to delayed treatment [4, 5]. In addition, the residual pleural hypertrophy (RPT) after treatment is quite common, affecting up to 50% of the total patients. In clinical practice, there are often TPE patients with pleural hypertrophy who suffer from chest collapse on the affected side, resulting in pulmonary restrictive ventilation disorders. Therefore, the prevention and early treatment of RPT is of great significance for the long-term recovery of the patient’s quality of life and work ability. In recent years, research on the treatment of RPT with urokinase injection has been drawing increasing attention. In such a context, this study aimed to conduct a meta-analysis on the efficacy of UK in the treatment of TPE, in order to clarify the therapeutic effect of UK on TPE patients. The studies included in this meta-analysis were randomized controlled trials that were identified from a comprehensive literature search across multiple databases according to the inclusion criteria established based on the TPE Diagnosis and Treatment Guidelines of China [6].

## Materials and methods

### Search strategy

The PubMed, CBM, EMbase, CNKI, Wanfang, Web of Science, and Cochrane Library databases were searched for RCTs related to the UK treatment for TPE that were publicly published from 2000 to 2023. The literature search was carried out by combining subject words and keywords. Specifically, the English search terms include “Tuberculous Pleurisies”, “Tuberculous Pleural Effusion”, “Urokinase”, and “RCT”. We have used corresponding keywords in the Chinese database. Taking CBM as an example, the detailed search strategy is shown in Table 1.

**Table 1:**
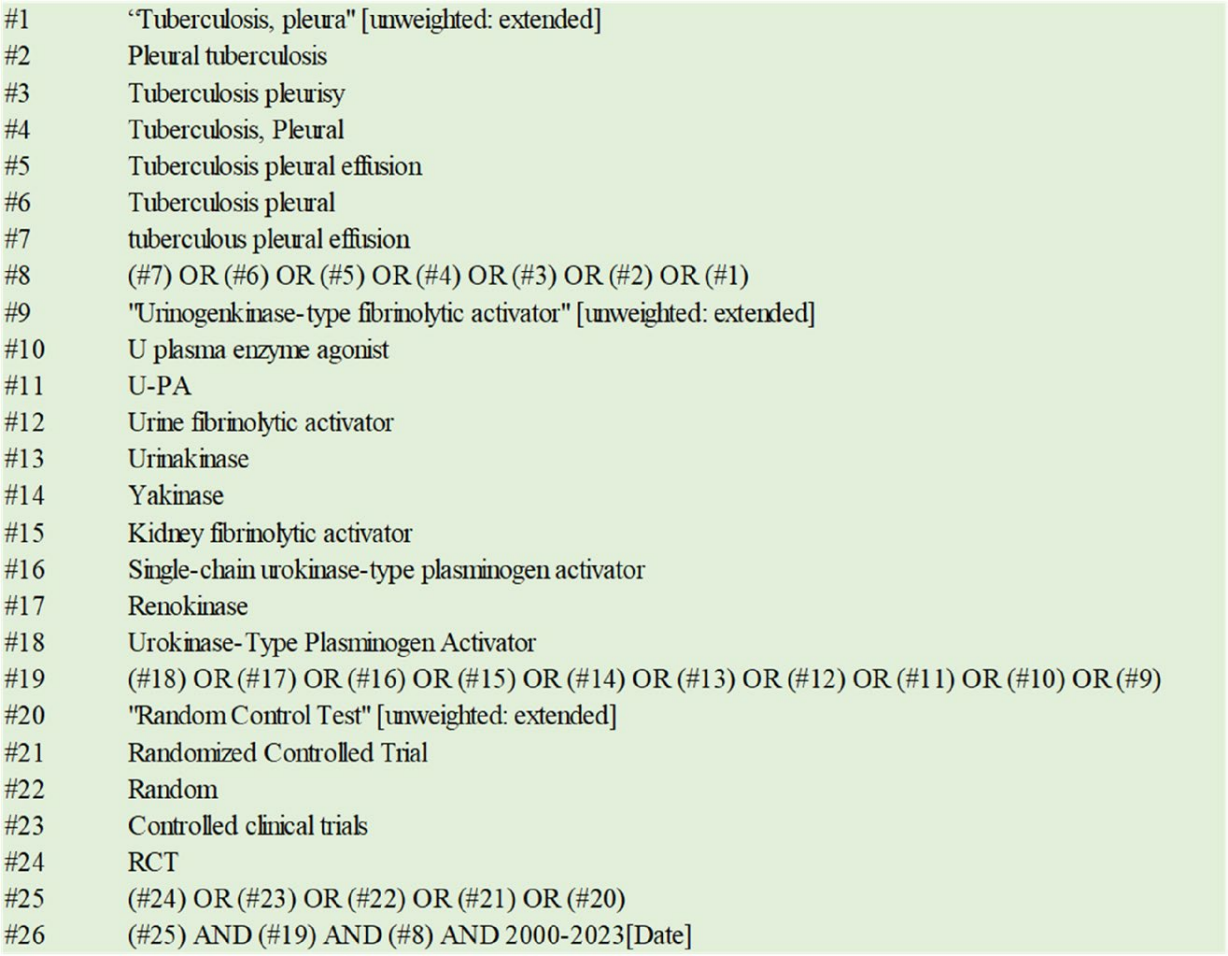
Search strategy of CBM

### Inclusion and exclusion criteria

#### Inclusion criteria

1. Participant:Patients with clinical symptoms and imaging diagnosis who meet the diagnostic criteria for tuberculosis pleuritis in the Guidelines for Primary Diagnosis and Treatment of Tuberculosis (2018) or in Internal Medicine [7, 8].
2. Intervention:Routine anti-tuberculosis therapy + thoracic puncture drainage or thoracic tube drainage + intrapleural injection of UK;
3. Comparison:Routine anti-tuberculosis therapy + thoracic puncture drainage or thoracic tube drainage ± intrapleural injection of an equal amount of 0.9% sodium chloride
4. Outcome:Absorption time of pleural effusion, residual pleural thickness, pleural drainage volume, FEV1% pred, and FVC% pred.
5. Study Design:RCT
6. All subjects in the experimental had no contraindications for the use of UK, such as abnormal coagulation function, hypersensitivity to UK, or history of hemorrhagic diseases within the past month.

#### Exclusion criteria

1. Non-cross-sectional studies, etc.;
2. Abstracts, lectures, reviews, repetitive reports, studies with incomplete clinical information, studies with incomplete data, studies in languages other than Chinese and English;
3. Non-tuberculous pleural effusion (e.g.hemothorax, non-tuberculous empyema, pleural effusion caused by other reasons);
4. Studies involving combined intrathoracic injection of drugs that may affect the efficacy evaluation of UK, such as heparin and hormones;
5. Studies whose data could not be utilized due to the fact that the data did not match the efficacy indicators in the inclusion criteria, and studies that did not clearly describe the experimental group and the control group.

### Outcome indicators

Absorption time of pleural effusion, residual pleural thickness, pleural drainage volume, FEV1% pred, and FVC% pred.

### Literature screening and data extraction

**Fig. 1:**
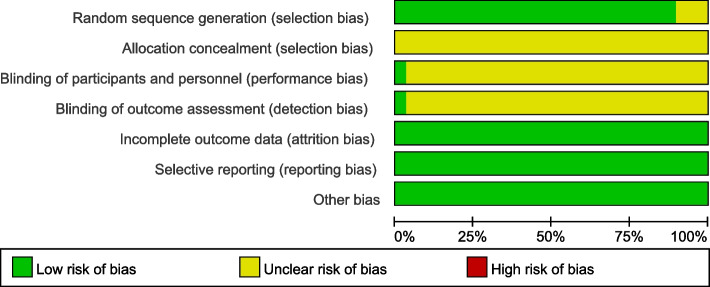
Risk of bias graph. The vertical axis of the figure is the risk assessment entry, and the horizontal axis is the percentage of “yes”, “no”, and “unclear” in the evaluation entry

**Fig. 2:**
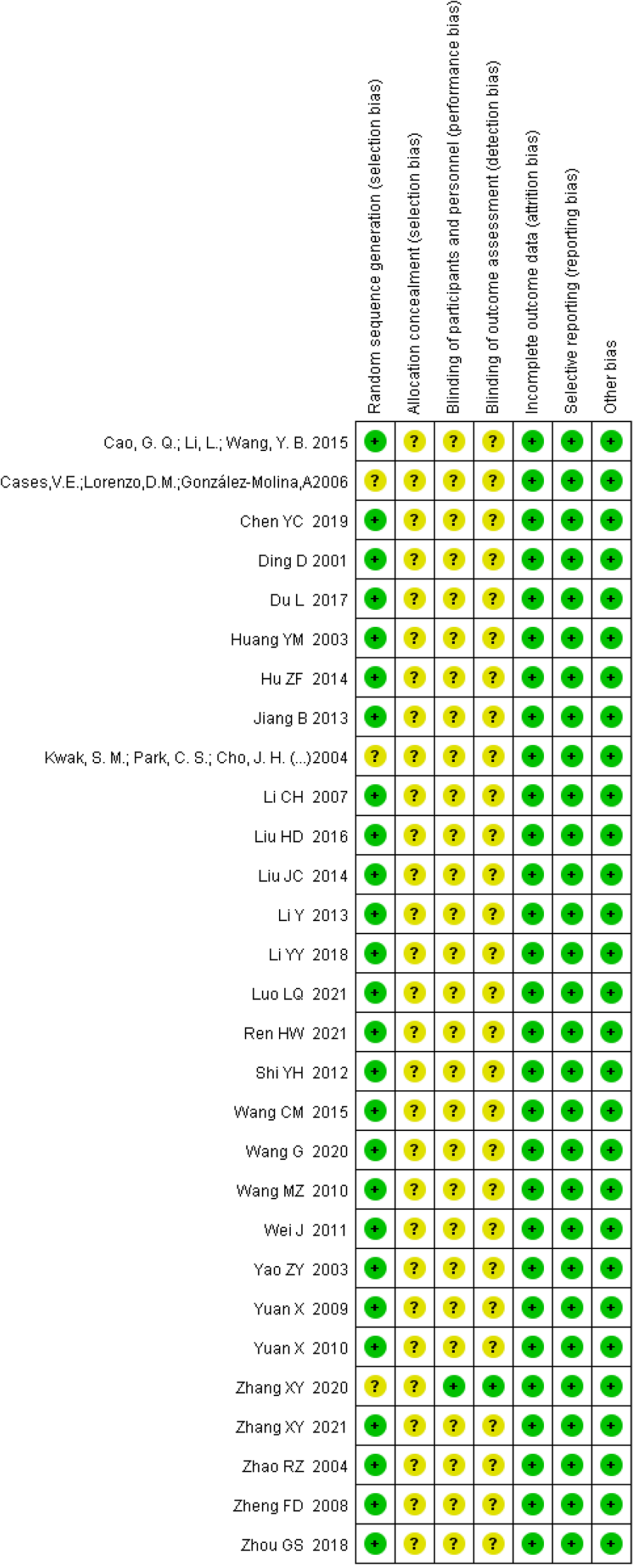
Risk of bias summary

The titles and abstracts of the preliminarily-retrieved studies from literature search were independently reviewed by two researchers. After excluding studies that were obviously irrelevant, the full texts of the remaining studies were examined and cross-checked by these two researchers for further screening. Disagreements, if any, were resolved by discussing with a third researcher. The study quality was evaluated by the Jadad scale method, where a score of 1-3 indicates low-quality and a score of 3-5 indicates high-quality [9] . The data of interest were extracted using a self-developed table, mainly including the basic study information, the baseline characteristics of study subjects, intervention measures, and outcome indicators. The bias risk ratio chart and the quality evaluation summary of the 29 included studies are shown respectively in Figs. 1 and 2 [4, 5, 10–36] .

### Statistical methods

The Review Manager 5.4.1 software was used for data processing and analysis. Continuous variables were represented by mean difference (MD) and the corresponding 95% CI [37]. When *P* > 0.05, it indicated no statistically significant heterogeneity between studies, and a fixed effects model was used for meta-analysis. When *P* < 0.05, heterogeneity between studies was confirmed. Accordingly, the sources of heterogeneity were analyzed. If there was no significant clinical heterogeneity between studies, a random effects model was used for combined analysis, and the results were explained and discussed. After combined analysis, *P* < 0.05 indicated a statistically significant difference [38]. When there was significant clinical and statistical heterogeneity in the results of the included studies, only descriptive analysis was performed. The funnel plot was used to analyze possible publication bias. If the plot was symmetrical, it indicated no publication bias; if the plot was asymmetrical, it indicated the possible existence of publication bias.

## Results

### Literature search results

**Fig. 3:**
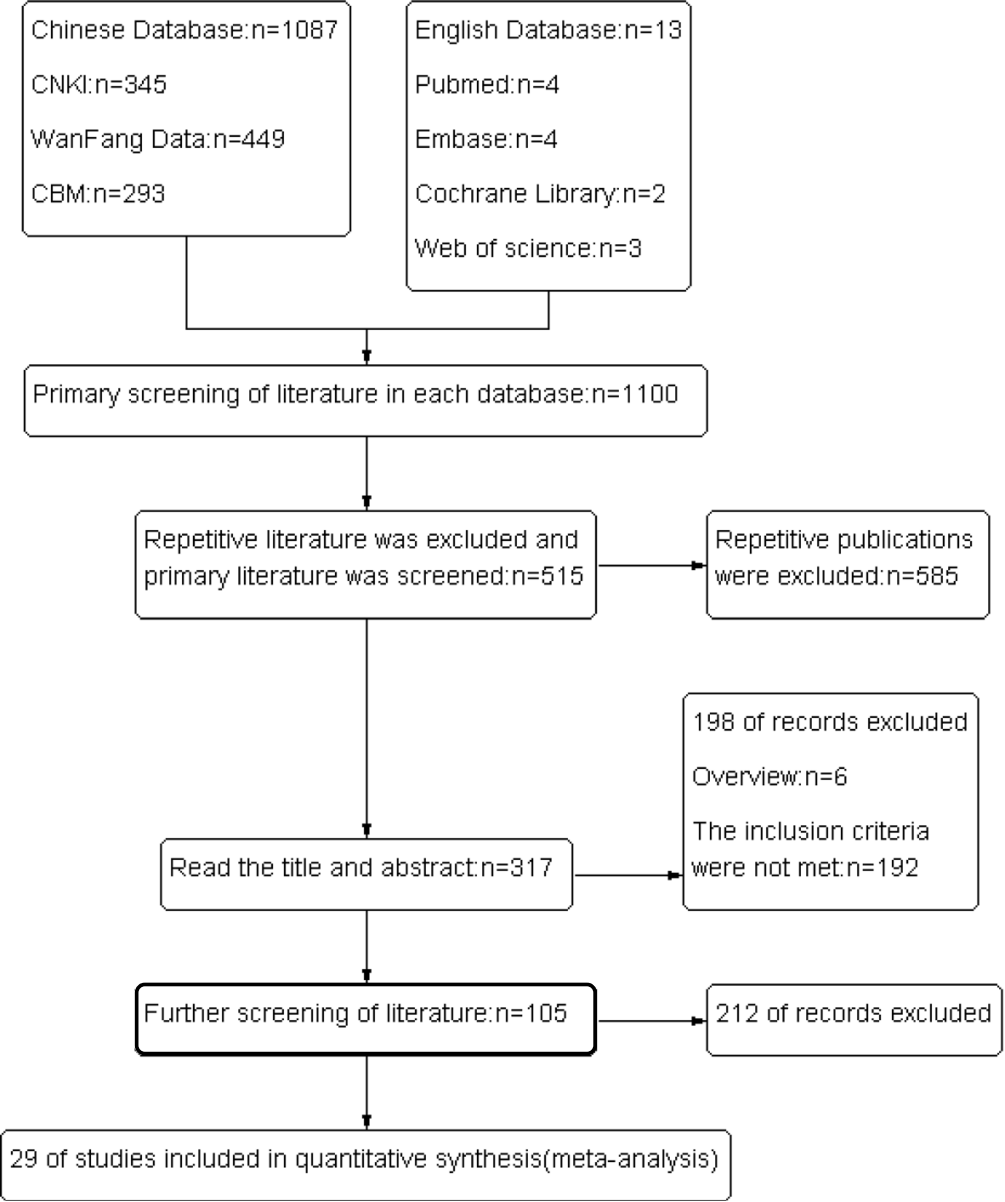
Study Flow Diagram

A total of 1087 Chinese studies and 13 English studies were retrieved from the preliminary literature search. After screening the titles and abstracts and excluding reviews and non-clinical studies, 317 articles were identified for full-text review and 105 articles met our inclusion and exclusion criteria. Further, through quality evaluation screening, 29 RCTs were finally included in our meta-analysis, covering a total of 2903 TPE patients (1459 in the UK treatment group and 1444 in the control group). The literature screening process and the search results are shown in Fig. 3. Basic Characteristics (Table 2) and Bias Risk Evaluation Results (Table 3).

**Table 2:** Basic characteristics of included studies

**Author**	**Experimental Group**	**Control Group**	**Male/Female Ratio**		**Age (years)**		**Anti-tuberculosis**	**UK Dose**	**Drainage Method**		**Outcomes**
			**Experimental Group**	**Control Group**	**Experimental Group**	**Control Group**	**Treatment**	**(10,000U)**	**Experimental Group**	**Control Group**	
Ren HW [4]	205	205	129/76	118/87	16～76	16～74	3HRZE/6HR	10	Puncture and Catheter Drainage	Puncture and Catheter Drainage	1.3.5
Zhang XY [5]	30	30	18/12	19/11	27～80	29～75	HRZE	10	Puncture and Catheter Drainage	Thoracentesis	1.3
Luo LQ [10]	43	43	28/15	25/17	22～58	25～60	2HRZE/10HRE	20	Puncture and Catheter Drainage	Puncture and Catheter Drainage	1.4.5
Wang G [11]	57	55	69/43 (There was no distinction between experimental and control groups)		15～59 (There was no distinction between experimental and control groups)		HRZE	10	Central venous catheter drainage	Puncture and Catheter Drainage	3
Zhang XW [12]	18	18	9/9	10/8	46～77	45～78		10	Central venous catheter drainage	Thoracentesis	1.2
Chen YC [13]	30	30	15/15	16/14	36.4 ± 12.7	35.5 ± 13.2	2HRZE/4HR	10	Central venous catheter drainage	Central venous catheter drainage	1.2.3.5
Li YY [14]	50	50	31/19	32/18	21～64	21～64	2HRZE/10HRE	10	Puncture and Catheter Drainage	Puncture and Catheter Drainage	1.2.3
Zhou GS [15]	59	55	31/28	29/26	23～76	21～79	2HRZ/4HR	20	Puncture and Catheter Drainage	Thoracentesis	1.2.3.4.5
Du L [16]	20	20	23/17 (There was no distinction between experimental and control groups )		17-57(There was no distinction between experimental and control groups)			3～5	Thoracentesis	Thoracentesis	1.2
Liu HD [17]	30	30	18/12	17/13	18～74	19～72	2HRZE/6HRE	10	Puncture and Catheter Drainage	Puncture and Catheter Drainage	1.2.3
Cao, G.Q.; Li, L.; Wang, Y.B. [18]	86	85						2～6	Puncture and Catheter Drainage	Puncture and Catheter Drainage	4
Wang CM [19]	50	50	32/18	30/20	17～65	16～62		10	Puncture and Catheter Drainage	Thoracentesis	2.3
Liu JC [20]	88	88	47/41	46/42	24～56	23～54	HRZE	10	Puncture and Catheter Drainage	Thoracentesis	1.2
Hu ZF [21]	42	42	62/22 (There was no distinction between experimental and control groups)		18～68 (There was no distinction between experimental and control groups)			10	Thoracentesis	Thoracentesis	1
Jiang B [22]	20	20	There was no significant difference in age, sex, course of disease and pulmonary function between the two groups (*P* > 0.05)					10	Thoracentesis	Thoracentesis	2.3
Li Y [23]	30	30	42/18 (There was no distinction between experimental and control groups)		18～65 (There was no distinction between experimental and control groups)		2HRZ/4HR	10	Puncture and Catheter Drainage	Puncture and Catheter Drainage	1.2.3
Shi YH [24]	130	130	62/68	74/56	17～85	16～70		10	Thoracentesis	Thoracentesis	1.2
Wei J [25]	40	40	24/16	22/18	18～53	17～55	3HRZ/6HR	10	Central venous catheter drainage	Thoracentesis	2.3
Wang MZ [26]	40	40	28/12	27/13	36.7±20.5	35.6 ± 19.7	2HRZE/4HR	10	Central venous catheter drainage	Thoracentesis	1.2
Yuan X [27]	36	36	26/10	24/12	18～56	17～59		10	Puncture and Catheter Drainage	Thoracentesis	1.2.3
Yuan X [28]	139	139	72/67	70/69	17～85	16～70		10	Puncture and Catheter Drainage	Thoracentesis	1.2
Zheng FD [29]	30	30	28/32 (There was no distinction between experimental and control groups)		16～62 (There was no distinction between experimental and control groups)		2SHRZ/4HR	20	Central venous catheter drainage	Thoracentesis	2.3
Li CH [30]	40	36	28/12	27/9	32.3 ± 7.2	35.8 ± 6.3	2HRZE/6HR	10	Central venous catheter drainage	Central venous catheter drainage	1.3
Cases, V.E.; Lorenzo, D.M.; Gonzdlez-Mlina, A [31]	12	17	21/8 (There was no distinction between experimental and control groups)					12.5	Puncture and Catheter Drainage	Puncture and Catheter Drainage	1.3
Kwak, S. M.; Park, C.S.; Cho, J.H [32]	21	22	15/6	12/10	30.6 ± 7.8	29.9 ± 10.0	2HRZE/4HRE	10	Puncture and Catheter Drainage	Puncture and Catheter Drainage	1.3
Zhao RZ [33]	34	35	23/11	22/13	16～51	19～57	2HRS/4HR	10	Thoracentesis	Thoracentesis	2.3
Yao ZY [34]	19	13	There was no significant difference in sex, age, course of disease, ESR and volume of pleural fluid before injection between the two groups (*P* > 0.05)				2HRZS/6HR	10	Puncture and Catheter Drainage	Puncture and Catheter Drainage	1.4.5
Huang YM [35]	20	20	9/11	11/9	41.8 ± 9.0	37.6 ± 10.6	2HRS/4HR	25	Central venous catheter drainage	Central venous catheter drainage	1.2.3
Ding D [36]	42	39	23/19	25/14	15～55	13～50	2HRS/4HR	10	Thoracentesis	Thoracentesis	1.3

1. Drainage volume of pleural effusion (ml) 2. Absorption time of pleural effusion (d) 3. Residual pleural thickness (RPT) (mm) 4. FVC%pred after treatment 5. FEV1%pred after treatment

**Table 3:** Results of risk of bias assessment

Author Method	Year	Random	Allocation Hidden	Blind	Selective Reporting Of Research Findings	Integrity Resulting Of The Other Data Of	Sources Bias
Ren HW	2021	Random Number Table	Unclear	Undlear	Not	Not	Not
Zhang XY	2021	Random Number Table	Unclear	Unclear	Not	Not	Not
Luo LQ	2021	Random Number Table	Unclear	Unclear	Not	Not	Not
Wang G	2020	Random Number Table	Unclear	Unclear	Not	Not	Not
Zhang XW	2020	Just Mention Random	Unclear	Completely Double Blind	Not	Not	Not
Chen YC	2019	Random Number Table	Unclear	Unclear	Not	Not	Not
Li YY	2018	Random Number Table	Unclear	Unclear	Not	Not	Not
Zhou GS	2018	Random Number Table	Unclear	Unclear	Not	Not	Not
Du L	2017	Random Number Table	Unclear	Unclear	Not	Not	Not
Liu HD	2016	Random Number Table	Unclear	Unclear	Not	Not	Not
Cao.G.O:Li.L;Wang Y.B.	2015	Random Number Table	Unclear	Unclear	Not	Not	Not
Wang CM	2015	Random Number Table	Unclear	Unclear	Not	Not	Not
Liu JC	2014	Random Number Table	Unclear	Unclear	Not	Not	Not
Hu ZF	2014	Random Number Table	Unclear	Unclear	Not	Not	Not
Jiang B	2013	Random Number Table	Unclear	Unclear	Not	Not	Not
Li Y	2013	Drawing of lots	Unclear	Unclear	Not	Not	Not
Shi YH	2012	Random Number Table	Undlear	Undlear	Not	Not	Not
WeiJ	2011	Random Number Table	Unclear	Unclear	Not	Not	Not
Wang MZ	2010	Random Number Table	Unclear	Unclear	Not	Not	Not
YuanX	2010	Random Number Table	Unclear	Unclear	Not	Not	Not
YuanX	2009	Random Number Table	Undlear	Unclear	Not	Not	Not
Zheng FD	2008	Random Number Table	Undear	Undlear	Not	Not	Not
Li CH	2007	Drawing of lots	Unclear	Unclear	Not	Not	Not
Cases,V.E.;Lorenzo,D.M. González-Molina,A	2006	Just Mention Random	Undear	Unclear	Not	Not	Not
Kwak, S.M.; Park,C.S.; Cho, J. H.	2004	Just Mention Random	Unclear	Undlear	Not	Not	Not
Zhao RZ	2004	Random Number Table	Unclear	Unclear	Not	Not	Not
Yao ZY	2003	The envelope drawing of lots	Unclear	Unclear	Not	Not	Not
Huang YM	2003	Drawing of lots	Unclear	Unclear	Not	Not	Not
Ding D	2001	Coin Toss	Unclear	Unclear	Not	Not	Not

### Efficacy analysis

#### Absorption time of pleural effusion

**Fig. 4:**
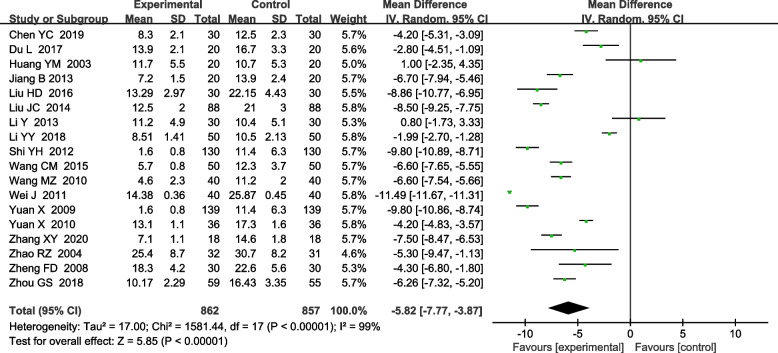
Meta-analysis forest plot of pleural effusion absorption time

For the analysis of absorption time of pleural effusion, 18 RCTs were included. The heterogeneity test indicated the existence of heterogeneity among included studies (x^2^ = 1581.44, I^2^ = 99%, *P* < 0.00001), so a random effects model was used for combined analysis. It was found that there was a statistically significant difference between the treatment group and the control group [MD-5.82, 95%CI (−7.77, −3.87); *P* < 0.00001], suggesting that the treatment group was superior to the control group in reducing the absorption time of pleural effusion (Fig. 4).

#### Residual pleural thickness after treatment

**Fig. 5:**
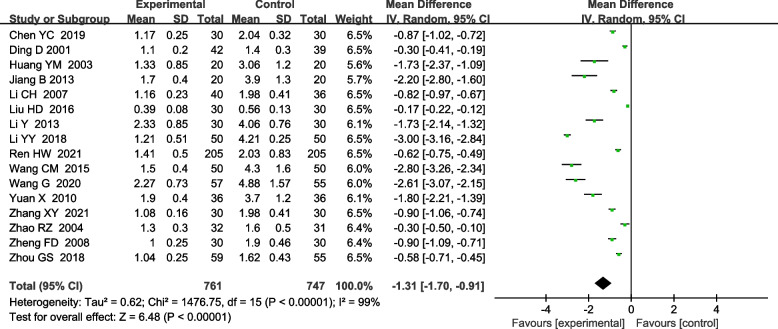
Meta-analysis forest plot of residual pleural thickness

A total of 16 RCTs reported the effect of UK on the pleural thickness [4, 5, 11, 13–15, 17, 19, 22, 23, 27, 29, 30, 33, 35, 36]. The heterogeneity test indicated the existence of heterogeneity among included studies (x^2^ = 1476.75, I^2^ = 99%, *P* < 0.00001)], so a random effects model was used for combined analysis. It was found that there was a statistically significant difference between the treatment group and the control group [MD-1.31, 95%CI (−1.70, −0.91); *P*<0.00001], suggesting that the treatment group was superior to the control group in reducing pleural thickness (Fig. 5).

#### Pleural effusion drainage volume

**Fig. 6:**
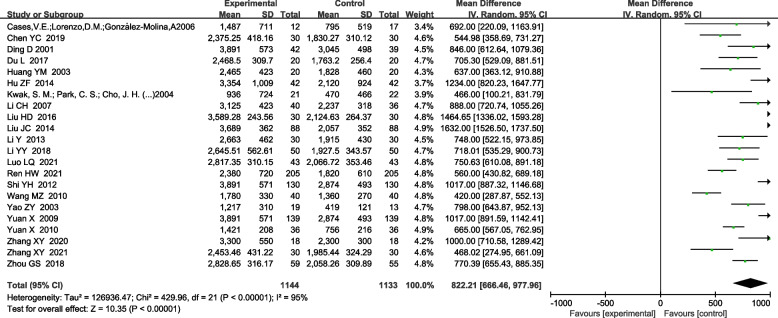
Forest plot of meta-analysis for comparison of drainage volume of pleural effusion

A total of 22 RCTs reported the effect of UK on the pleural effusion drainage volume [4, 5, 10, 12–17, 20, 21, 23, 24, 26–28, 30–32, 34–36]. The heterogeneity test indicated the existence of heterogeneity among included studies (x^2^ = 429.96, I^2^ = 95%, *P* < 0.00001), so a random effects model was used for combined analysis. Compared with the control group, the pleural effusion drainage volume was obviously increased in the treatment group and the difference was statistically significant [MD 822.81, 95%CI (666.46, 977.96); *P*<0.00001] (Fig. 6).

#### FVC% pred after treatment and FEV1% pred after treatment

**Fig. 7:**
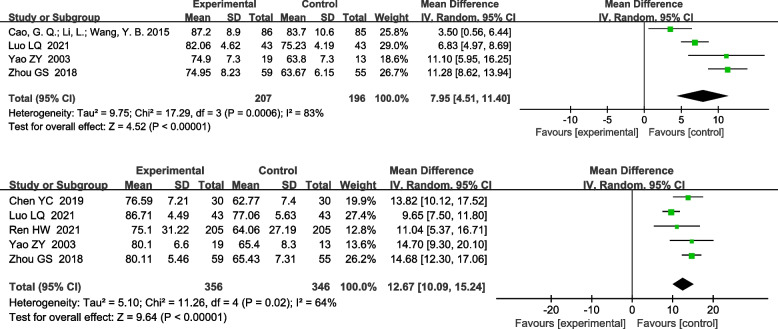
Forest plot of meta-analysis comparing FVC% pred and Forest plot of meta-analysis comparing Fev1% pred

A total of 4 RCTs reported the effect of UK on the FVC% pred [10, 15, 18, 34]. The heterogeneity test indicated the existence of heterogeneity among included studies (x^2^ = 17.29, I^2^ = 83%, *P* = 0.0006), so a random effects model was used for combined analysis. It was found that there was a statistically significant difference between the treatment group and the control group [MD 7.95, 95%CI (4.51, 11.40); *P*<0.00001], suggesting that UK was able to significantly improve. A total of 5 RCTs reported the effect of UK on the FEV1% pred [4, 10, 13, 15, 34]. The heterogeneity test indicated the existence of heterogeneity among included studies (x^2^ = 11.26, I^2^ = 64%, *P* = 0.02), so a random effects model was used for combined analysis. It was found that there was a statistically significant difference between the treatment group and the control group [MD 12.67, 95%CI (10.09, 15.24); P<0.00001], suggesting that UK was able to significantly improve lung function (Fig. 7).

### Subgroup analysis

Subgroup analyses were conducted in terms of UK dosage among the 29 included RCTs. Specifically, there were 2 articles with UK dosage< 100,000 IU [16, 18] 22 articles with UK dosage = 100,000 IU [4, 5, 11–14, 17, 19–28, 30, 32–34, 36], 1 article with UK dosage = 125,000 IU [31], 3 articles with UK dosage = 200,000 IU [10, 15, 29], and 1 article with UK dosage = 250,000 IU [35].

#### UK on FEV1% pred

**Fig. 8:**
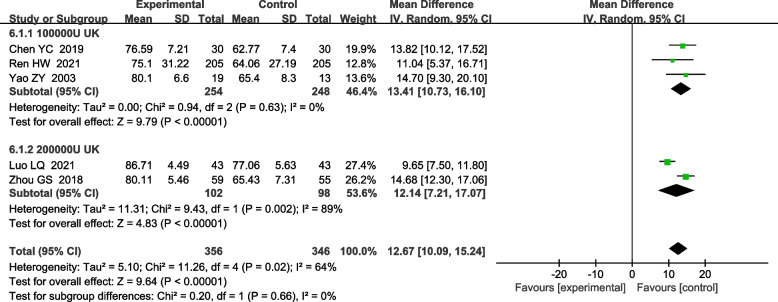
Forest plot for meta-analysis of Fev1% pred in UK subgroups compared with controls

For the subgroup analysis of UK on the FEV1% pred, there were 2 articles with UK dosage of 200,000 IU [10, 15], and 3 articles with UK dosage of 100,000 IU [5, 13, 34]. Both subgroups were analyzed using a random effects model. The differences in the 200,000 IU subgroup [MD 12.14, 95%CI (7.21, 17.07); *P* < 0.0001] and in the 100,000 IU subgroup [MD 13.41, 95%CI (10.73, 16.10); *P* < 0.00001] were both statistically significant (Fig. 8).

#### UK on pleural thickness

**Fig. 9:**
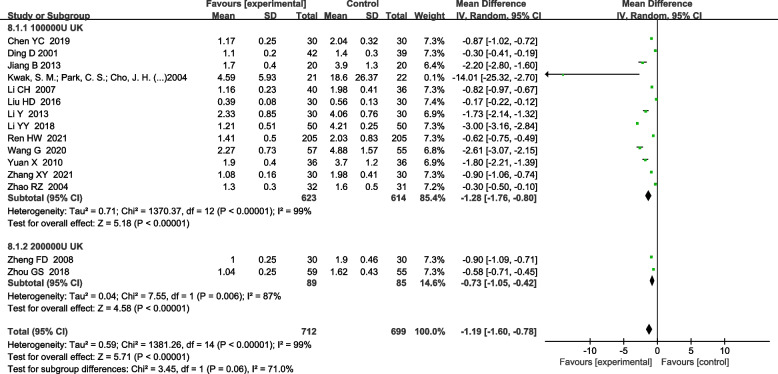
Effects of UK subgroup and control group on pleural thickness

For the subgroup analysis of UK on the pleural thickness, there were 13 articles with UK dosage of 100,000 IU [4, 5, 11, 13, 14, 17, 22, 23, 27, 30, 32, 33, 36], and 2 articles with UK dosage of 200,000 IU [15, 29]. Both subgroups were analyzed using a random effects model. Similarly, the differences in the 200,000 IU subgroup [MD-0.73 (− 1.05, − 0.42), *P* < 0.0001] and in the 100,000 IU subgroup [MD-1.28 (− 1.76, − 0.80), *P* < 0.0001] were both statistically significant (Fig. 9).

#### UK on pleural effusion drainage volume

**Fig. 10:**
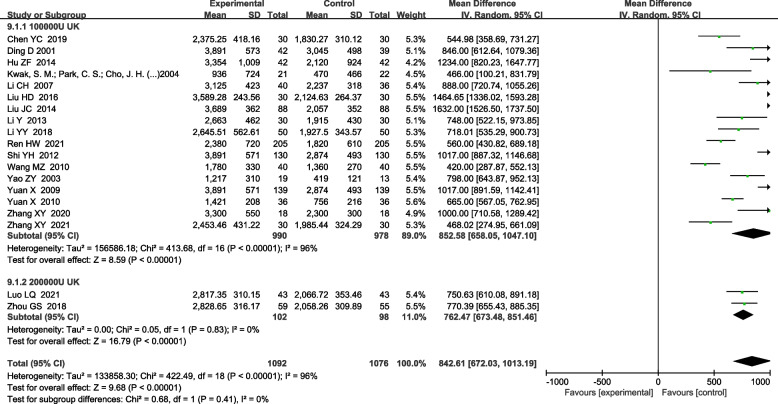
Effect of UK subgroup and control group on drainage volume of pleural effusion

For the subgroup analysis of UK on the pleural effusion drainage volume, there were 17 articles with UK dosage of 100,000 IU [4, 5, 12–14, 17, 20, 21, 23, 24, 26–28, 30, 32, 34, 36]. The heterogeneity test indicated the existence of heterogeneity among these studies (χ^2^ = 413.68, *P* < 0.0001, I^2^ = 96%). Accordingly, a random effects model was used for combined analysis, and the difference was found to be statistically significant [MD 852.58 (658.051047.10), *P* < 0.0001]. In addition, there were 2 articles with UK dosage of 200,000 IU [10, 15], including 102 subjects in the UK treatment group and 98 subjects in the control group. Th heterogeneity test indicated no heterogeneity among these two studies (χ^2^ = 0.05, *P* = 0.83, I^2^ = 0%). A random effects model was used for combined analysis, and the difference was also statistically significant [MD 762.47 (673.48851.46), *P* < 0.0001] (Fig. 10).

### Publication Bias

**Fig. 11:**
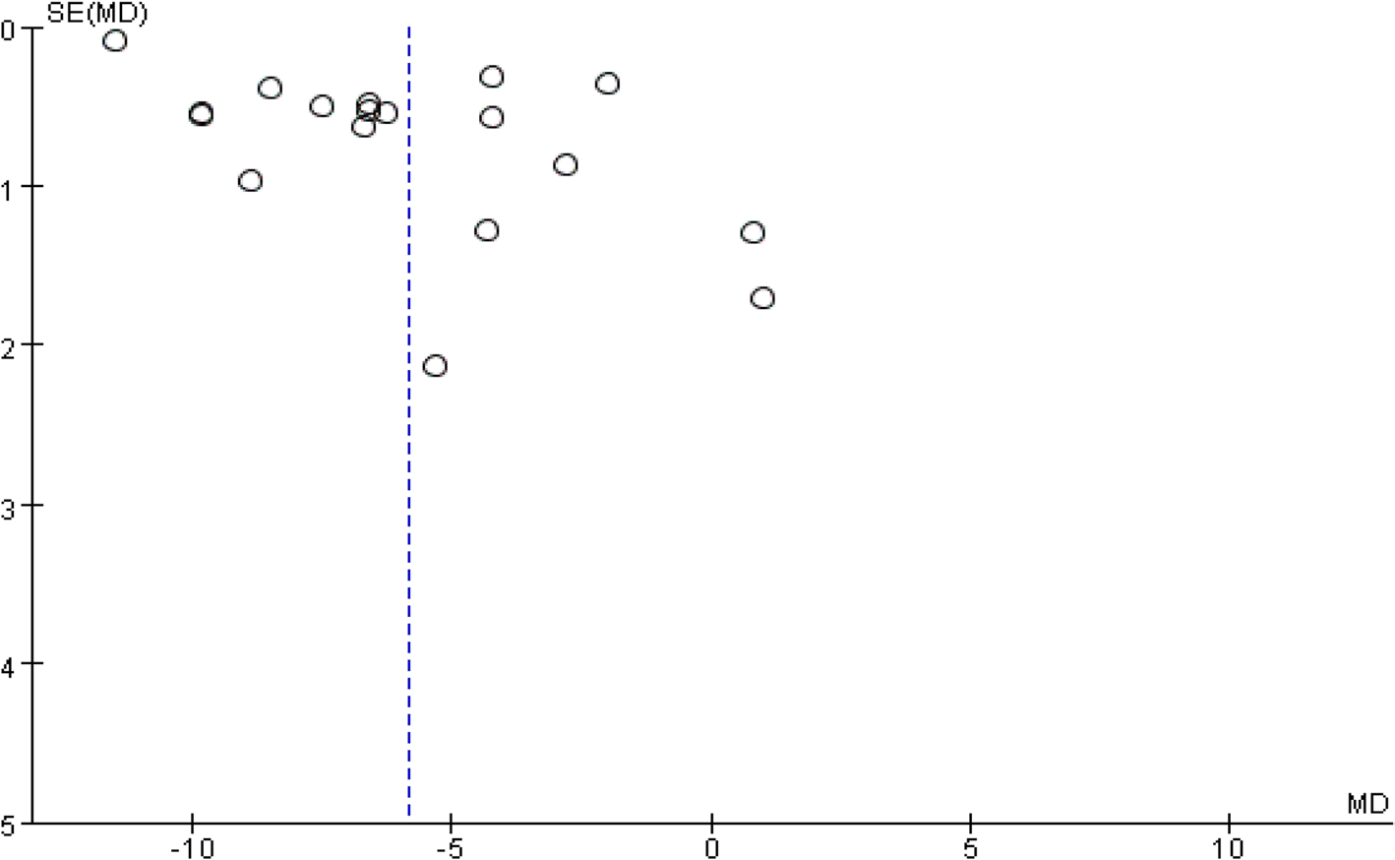
Pleural effusion time to complete absorption, funnel plot

**Fig. 12:**
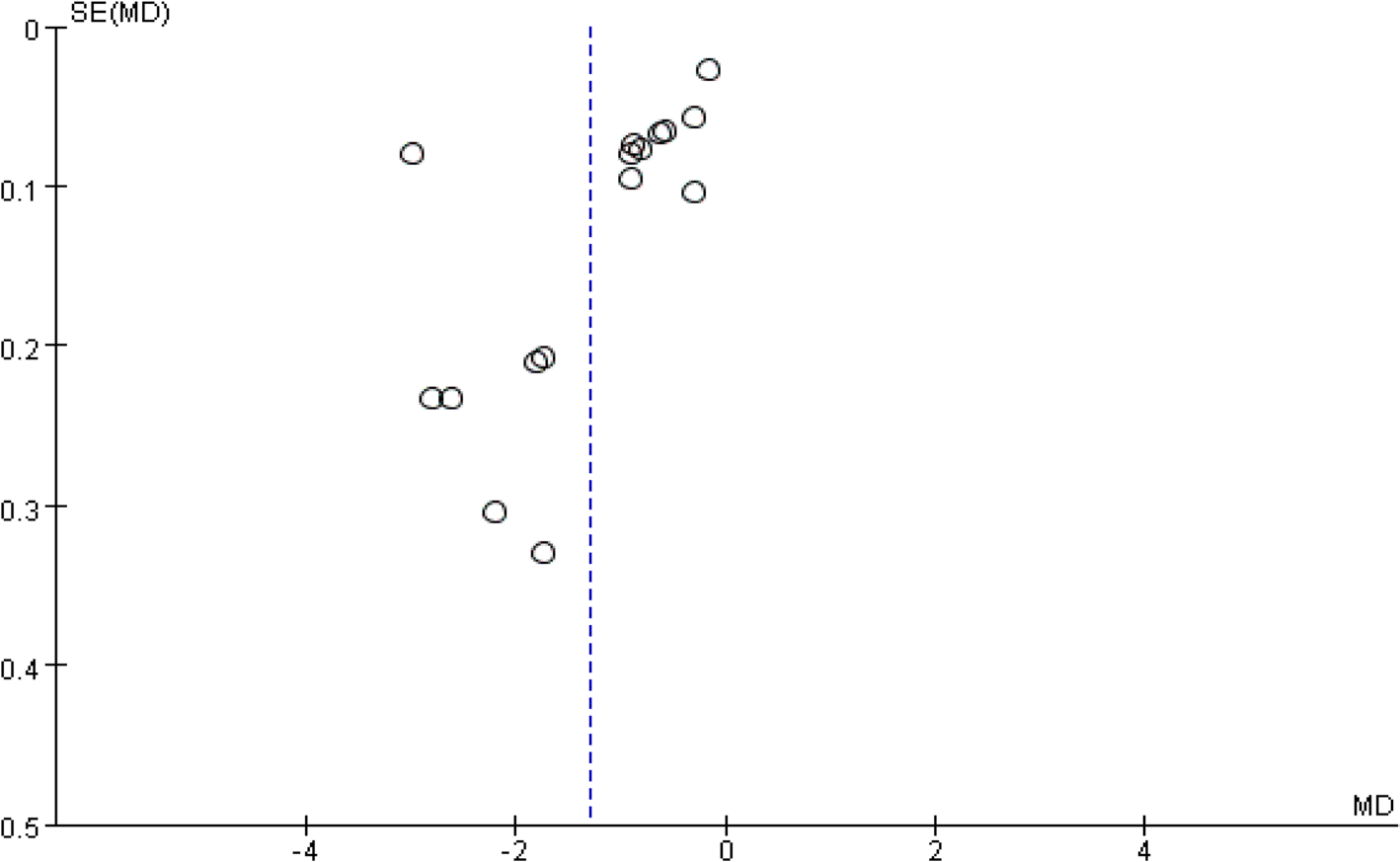
Residual pleural thickness, funnel plot

**Fig. 13:**
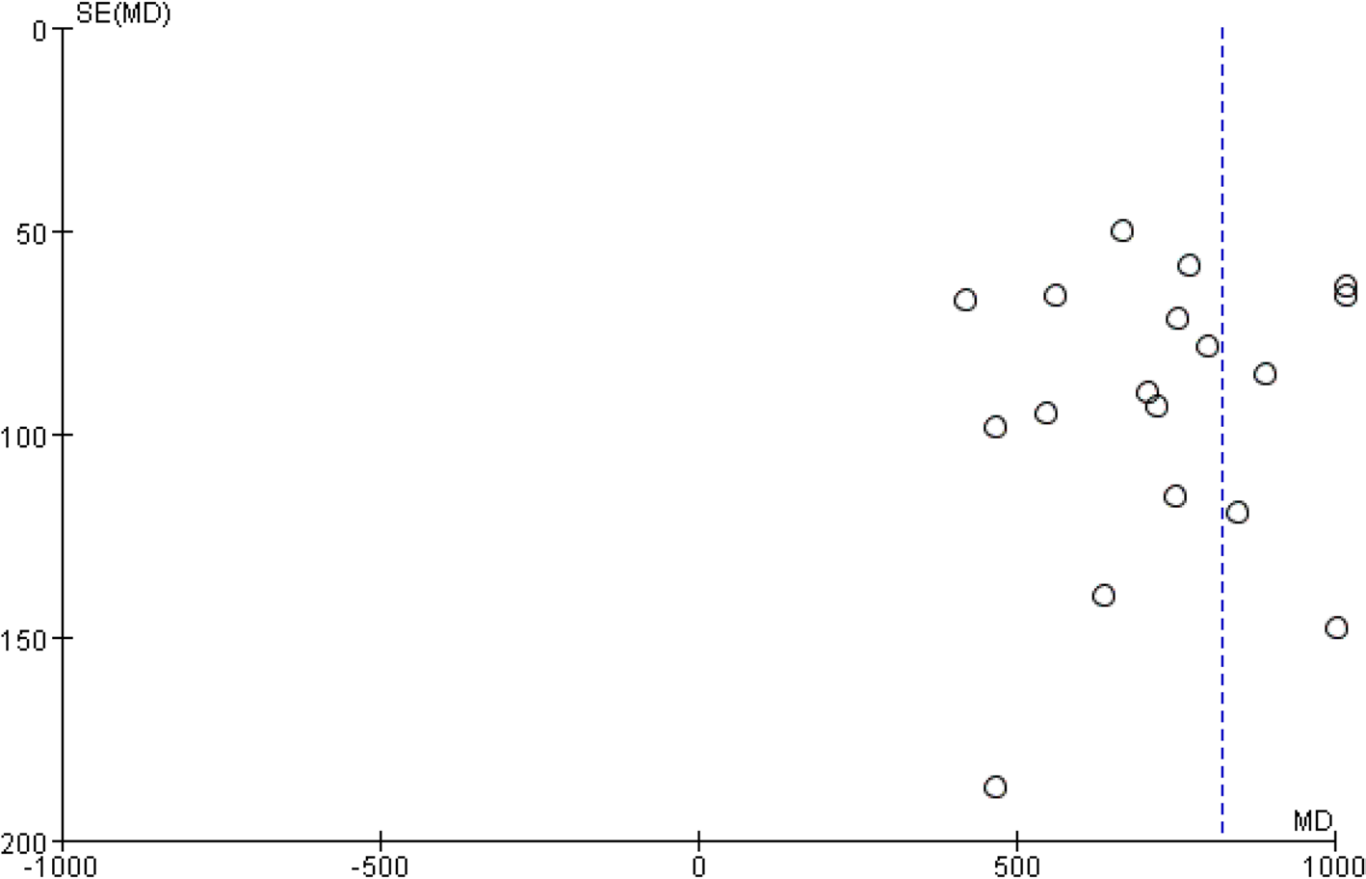
Volume of pleural effusion drainage, funnel plot

Funnel plots were drawn with the sample size as the vertical axis and the effect size as the horizontal axis. It was found that the funnel plots for the complete absorption time of pleural effusion (Fig. 11), the residual pleural thickness (Fig. 12) and the pleural thickness (Fig. 13) all appeared to be asymmetric, indicating the presence of publication bias [38]. As only a small number of studies were included in the subgroup analyses for FVC% pred and FVE1% pred, funnel plot analysis was not conducted.

## Discussion

Our meta-analysis of 29 RCTs showed that the UK treatment group had a significant increase in the pleural effusion drainage volume and lung function (FEV1% pred, FVC% pred), and a significant decrease in the pleural thickness and absorption time of pleural effusion. All these differences were statistically significant (*P* < 0.05), suggesting that the combined UK therapy could significantly increase the pleural effusion drainage volume, shorten the absorption time of pleural effusion, reduce pleural thickness, and improve lung function (FEV1% pred, FVC% pred). However, obvious heterogeneity was observed in the results of these 5 indicators, which may be related to the length of the patient’s disease course, the UK dosage, and the injection method. To reduce the possibility of analysis bias, we conducted subgroup analyses for various indicators in terms of the UK dosage. It was found that the results for the decrease in pleural thickness, increase in pleural effusion drainage volume, and improvement in FEV1% pred were similar to those of the overall analysis, and the differences between the treatment group and control group were statistically significant.

Tuberculous pleurisy is the extrapulmonary tuberculosis caused by the first invasion of tuberculous bacteria into the pleural cavity of human body. There are three ways for tuberculous bacteria to reach the pleural cavity, namely direct spread of lesions, lymphatic dissemination, and hematogenous dissemination [39]. At present, the main methods for treating TPE include routine anti-tuberculosis therapy, the use of adrenal cortical hormones, puncturing for drainage, thoracic intervention treatment, thoracoscopic local treatment, and surgical treatment [40]. After formal and comprehensive anti-tuberculosis treatment, the vast majority of TPE patients could recover. However, due to the high response of the pleura to tuberculosis toxin, it can easily cause exudation. Consequently, some patients may develop pleural effusion in a short period of time due to fibrin cell fragments and cellulose covering the surface of the pleura in the pleural fluid [41]. Meanwhile, the continuous production and excessive accumulation of pleural effusion can further lead to pleural adhesiveness thickening and increased compression on the lungs [42] thereby affecting the patient’s lung function and quality of life [43]. In clinical practice, the intrathoracic injection of hormones and anti-tuberculosis drugs can only reduce inflammatory exudation but not treat the already exuded fluid. An earlier study showed that plasminogen activator inhibitors (PAI) played a decisive role in the fibrinolytic level of pleural effusion, especially PAI-1, which might be related to tissue regeneration, repair and fibrosis development after pleural injury [44] .Pollack reported that UK could exert a good therapeutic effect when the formation of pleural fluid had not exceeded 6 weeks and the fibrins had not yet been widely deposited, adhered or separated [45]. Huang found that the intrathoracic injection of UK could effectively prevent and treat pleural hypertrophy and adhesion in clinical practice [46]. Zhang pointed out that the large amount of fibrin contained in TPE would lead to effusion thickening and generation of protein clots, which might induce the occurrence of multiple pathological processes such as multiloculated and pleural fibrosis [47]. In this regard, the plasmin activated by UK can crack the fibrin loculated in the pleural effusion, eliminate the blockage of the fiberloculated to the puncture needle or drainage tube, thus facilitating the drainage of pleural effusion [48]. The research by Lin showed that [49], after injection of UK, the pleura was significantly thinned and the cellulose deposition and loculated were significantly reduced compared with the situation after simple conventional anti-tuberculosis treatment. According to the above research results, UK has an obvious effect in the treatment of TPE.

The results of our meta-analysis suggest that the intrathoracic injection of UK is able to promote the absorption of pleural fluid and increase the pleural drainage volume for TPE patients, so as to exert a positive effect in reducing pleural thickness and improving lung function. This is consistent with the related reports at home and abroad [31, 32, 49–51], providing additional evidence for the therapeutic effect of UK on TPE. Compared with the meta-analysis conducted by Xia [52],we performed a comprehensive screening and quality evaluation on the retrieved studies from literature search. It was found that two included studies in Xia’s meta-analysis were questionable: the study by Li Shiying grouped the patients according to the sequence of hospitalization time; the study by Gao Chunrong grouped the patients according to the sequence of admission in terms of odd or even numbers, and the results were incomplete without any explanation on the reasons of missing data.

In summary, UK is more effective in treating TPE compared with the conventional anti-tuberculosis therapy alone. Specifically, it can increase the pleural drainage volume, reduce the residual pleural thickness, shorten the absorption time of pleural effusion, and improve lung function (FEV1% pred, FVC% pred). Our study supports that UK has good efficacy in the treatment of TPE and provides a useful reference for clinical practice.

However, some limitations should be highlighted: ① Our meta-analysis only included Chinese and English articles without searching studies in other languages; ② There were differences in terms of the conventional anti-tuberculosis treatment plan, the pleural effusion drainage method, the UK dosage, and the injection method among different studies, so the experimental results were subjected to bias to some extent; ③ The data provided by the included studies were limited, and the course of disease was not investigated; ④ Most of the included studies did not provide a specific description of the double blind methods implemented to the subjects, experimenters, and evaluators, resulting in an increased risk of implementation bias and a generally low Jadad score; ⑤ Most of the included studies had a small sample size, and there might be deviations between the results and the actual situation. Given the limitations of this study, our findings need to be further verified by more high-quality, large-scale clinical studies both domestically and internationally.

### Supplementary Information

**Additional file 1.**

**Additional file 2.**

## Data Availability

The datasets used and analysed during the current study available from the corresponding author on reasonable request.
