# Supplementary material for: Urokinase in the treatment of tuberculous pleurisy: a systematic review and meta-analysis
Source: BMC Infect Dis. 2024 Feb 24;24:258. doi: 10.1186/s12879-024-08975-0 (PMC10893646; doi:10.1186/s12879-024-08975-0)
Supplement: Supplementary file 1 — Additional file 1. [file 12879_2024_8975_MOESM1_ESM.docx]

| **Section and Topic** | **Item #** | **Checklist item** | **Location where item is reported** |
| --- | --- | --- | --- |
| **TITLE** | | |  |
| Title | 1 | Urokinase in the treatment of Tuberculous pleurisy: A systematic review and meta-analysis |  |
| **ABSTRACT** | | |  |
| Abstract | 2 | Objective :To evaluate the efficacy of urokinase treatment for tuberculous pleural effusion.  Methods: We searched Chinese biomedical literature database, WanFang data、CNKI 、PubMed 、EMbase、Web of Science and The Cochrane Library for the randomized controlled trials of urokinase treatment for tuberculous pleurisy from January 2000 to February 2023. Pleural tuberculosis, urokinase and randomized controlled trial were used as keywords. The eligible studies were meta-analyzed by using Revman 5.4.1: risk of bias was assessed, mean difference and 95% confidence interval were used for continuous variables, pooled studies were conducted using random-effects or fixed-effects models, forest plots were drawn to analyze efficacy, and funnel plots were drawn to discuss publication bias. Results:29 RCTs were included. The meta-analyzed results showed that, on the basis of routine anti-tuberculosis, comparison between the treatment group treated with urokinase and the control group treated with antituberculosis alone，the time of pleural effusion absorption [ MD-5.82, 95%CI (-7.77, -3.87); P＜0.00001] and the residual pleural thickness [ MD-1.31, 95%CI (-1.70, -0.91)；P＜0.00001], pleural effusion drainage volume [ MD 822.81, 95%CI(666.46,977.96);P＜0.00001] , FVC%pred [ MD 7.95, 95%CI (4.51,11.40) ; P＜0.00001] , FEV1%pred [ MD 12.67，95%CI (10.09,15.24); P＜0.00001] were significantly different.  Conclusion:The clinical effect of urokinase is better than that of antituberculous therapy alone: it can increase total pleural effusion, decrease residual pleural thickness, improve the pulmonary function, and shorten the time of pleural effusion absorption. |  |
| **INTRODUCTION** | | |  |
| Rationale | 3 | Tuberculous pleural effusion（TPE） is the most common infectious pleural disease and one of the major respiratory diseases in China.^1^ The global tuberculosis report 2022 shows that, an estimated 10.6 million people became ill with tuberculosis in 2021, and 1.6 million people died from tuberculosis in 2021, among which about 64,000 died in China.^2^ Tuberculous pleurisy is more prevalent in those countries with high prevalence of tuberculosis, and in China, tuberculous pleurisy accounts for about 50% of pleural effusion cases. ^3^ The traditional treatment for TPE is systemic anti-tuberculosis therapy combined with local fluid extraction, but many patients may easily develop pleural hypertrophy, adhesions, and encapsulated effusion due to delayed treatment. ^4,5^ In addition, the residual pleural hypertrophy (RPT) after treatment is quite common, affecting up to 50% of the total patients. In clinical practice, there are often TPE patients with pleural hypertrophy who suffer from chest collapse on the affected side, resulting in pulmonary restrictive ventilation disorders. Therefore, the prevention and early treatment of RPT is of great significance for the long-term recovery of the patient’s quality of life and work ability. In recent years, research on the treatment of RPT with UK injection has been drawing increasing attention. In such a context, this study aimed to conduct a meta-analysis on the efficacy of UK in the treatment of TPE, in order to clarify the therapeutic effect of UK on TPE patients. The studies included in this meta-analysis were randomized controlled trials（RCTs） that were identified from a comprehensive literature search across multiple databases according to the inclusion criteria established based on the TPE Diagnosis and Treatment Guidelines of China. ^6^ |  |
| Objectives | 4 | To evaluate the efficacy of urokinase treatment for tuberculous pleural effusion. |  |
| **METHODS** | | |  |
| Eligibility criteria | 5 | **Inclusion Criteria**  (1)Participant：Patients with clinical symptoms and imaging diagnosis who meet the diagnostic criteria for tuberculosis pleuritis in the Guidelines for Primary Diagnosis and Treatment of Tuberculosis (2018).^7^  (2)Intervention：Routine anti-tuberculosis therapy + thoracic puncture drainage or thoracic tube drainage + intrapleural injection of UK;  (3)Comparison：Routine anti-tuberculosis therapy + thoracic puncture drainage or thoracic tube drainage ± intrapleural injection of an equal amount of 0.9% sodium chloride  (4)Outcome：Absorption time of pleural effusion, residual pleural thickness, pleural drainage volume, FEV1% pred, and FVC% pred.  (5)Study Design：RCT  (6) All subjects in the experimental had no contraindications for the use of UK, such as abnormal coagulation function, hypersensitivity to UK, or history of hemorrhagic diseases within the past month.  **Exclusion Criteria**  (1) Non-cross-sectional studies, etc;  (2) Abstracts, lectures, reviews, repetitive reports, studies with incomplete clinical information, studies with incomplete data, studies in languages other than Chinese and English;  (3) Non-tuberculous pleural effusion (e.g., hemothorax, empyema, pleural effusion caused by other reasons);  (4) Studies involving combined intrathoracic injection of drugs that may affect the efficacy evaluation of UK, such as heparin and hormones;  (5) Studies whose data could not be utilized due to the fact that the data did not match the efficacy indicators in the inclusion criteria, and studies that did not clearly describe the experimental group and the control group. |  |
| Information sources | 6 | The PubMed, CBM, EMbase, CNKI, Wanfang, Web of Science, and Cochrane Library databases were searched for RCTs related to the UK treatment for TPE that were publicly published from 2000 to 2023. |  |
| Search strategy | 7 | The literature search was carried out by combining subject words and keywords. Specifically, the Chinese search terms include: “tuberculous pleurisy”“Tuberculous pleural effusion”“urokinase”“randomized controlled”; the English search terms include “Tuberculous Pleurisies”, “Tuberculous Pleural Effusion”, “Urokinase”, and “RCT”. Taking CBM as an example, the detailed search strategy is shown in Box 1. The full search strategy for all of the databases is provided in Attachment 2. |  |
| Selection process | 8 | The titles and abstracts of the preliminarily-retrieved studies from literature search were independently reviewed by two researchers. After excluding studies that were obviously irrelevant, the full texts of the remaining studies were examined and cross-checked by these two researchers for further screening. Disagreements, if any, were resolved by discussing with a third researcher. |  |
| Data collection process | 9 | The titles and abstracts of the preliminarily-retrieved studies from literature search were independently reviewed by two researchers. After excluding studies that were obviously irrelevant, the full texts of the remaining studies were examined and cross-checked by these two researchers for further screening. Disagreements, if any, were resolved by discussing with a third researcher. The study quality was evaluated by the Jadad scale method, where a score of 1-3 indicates low-quality and a score of 3-5 indicates high-quality. ^8^ The data of interest were extracted using a self-developed table. |  |
| Data items | 10a | We collected data on:  the outcome indicators:Absorption time of pleural effusion, residual pleural thickness, pleural drainage volume, FEV1% pred, and FVC% pred. |  |
|  | 10b | We collected data on:  the report:the basic study information(author, year, and source of publication);  the study:the baseline characteristics of study subjects,  the intervention:treatment plan, drainage method, dosage, and the injection method. |  |
| Study risk of bias assessment | 11 | The titles and abstracts of the preliminarily-retrieved studies from literature search were independently reviewed by two researchers. After excluding studies that were obviously irrelevant, the full texts of the remaining studies were examined and cross-checked by these two researchers for further screening. Disagreements, if any, were resolved by discussing with a third researcher. The study quality was evaluated by the Jadad scale method, where a score of 1-3 indicates low-quality and a score of 3-5 indicates high-quality. ^8^  The Review Manager 5.4.1 software was used for data processing and analysis. |  |
| Effect measures | 12 | Continuous variables were represented by mean difference (MD) and the corresponding 95% CI.^36^ |  |
| Synthesis methods | 13a | Considering the complexity of the outcome measures under investigation, we classified the outcome measures in the following categories: the time of pleural effusion absorption, residual pleural thickness, pleural water drainage flow, FEV 1% pred, and FVC% pred. We then organized the study according to the outcome measures included in the included studies. |  |
|  | 13b | When the individual data is missing, we chose to delete the individual data method without affecting the final data results. |  |
|  | 13c | The data of interest were extracted using a self-developed table, mainly including the basic study information, the baseline characteristics of study subjects, intervention measures, and outcome indicators.The basic characteristics of included studies are shown in table1.The eligible studies were meta-analyzed by using Revman 5.4.1: forest plots were drawn to analyze efficacy, and funnel plots were drawn to discuss publication bias. |  |
|  | 13d | The Review Manager 5.4.1 software was used for data processing and analysis. Continuous variables were represented by mean difference (MD) and the corresponding 95% CI.36 When P>0.05, it indicated no statistically significant heterogeneity between studies, and a fixed effects model was used for meta-analysis. When P<0.05, heterogeneity between studies was confirmed. Accordingly, the sources of heterogeneity were analyzed. If there was no significant clinical heterogeneity between studies, a random effects model was used for combined analysis, and the results were explained and discussed. After combined analysis, P<0.05 indicated a statistically significant difference.37 When there was significant clinical and statistical heterogeneity in the results of the included studies, only descriptive analysis was performed. |  |
|  | 13e | There was significant heterogeneity in the results of the 5 indicators in the study. We used subgroup analysis to explore the possible causes of heterogeneity among the results. We conducted a subgroup analysis of various measures of UK dose. |  |
|  | 13f | We pooled data using the random-effects model but we also analysed the fixed-efffect model to ensure robustness of the model chosen and susceotibility to outliers. |  |
| Reporting bias assessment | 14 | The funnel plot was used to analyze possible publication bias. If the plot was symmetrical, it indicated no publication bias; if the plot was asymmetrical, it indicated the possible existence of publication bias. |  |
| Certainty assessment | 15 | The credibility and utility of the pooled results of the meta-analysis are largely influenced by the quality of the included studies, so we assessed the quality of the included original studies using the Jada scale before analysis.(a score of 1-3 indicates low-quality and a score of 3-5 indicates high-quality. )^8^ |  |
| **RESULTS** | | |  |
| Study selection | 16a | The literature screening process and the search results are shown in Figure 3. |  |
|  | 16b | All the studies cited in this paper must meet the inclusion criteria, and if they do not meet the inclusion criteria, they will be excluded. |  |
| Study characteristics | 17 | The basic characteristics of the included studies are shown in Table 1. |  |
| Risk of bias in studies | 18 | The results of bias risk assessment are shown in Figure 1. |  |
| Results of individual studies | 19 | We classified the outcome measures in the following categories: the time of pleural effusion absorption, residual pleural thickness, pleural water drainage flow, FEV 1% pred, and FVC% pred. The results of each study are shown in Figures 4 to 13. |  |
| Results of syntheses | 20a | The bias risk ratio chart of the 29 included studies are shown respectively in Figure 1. ^4,5, 9-35^ |  |
|  | 20b | Results of all statistical syntheses:the time of pleural effusion absorption [ MD-5.82, 95%CI (-7.77, -3.87); P＜0.00001] and the residual pleural thickness [ MD-1.31, 95%CI (-1.70, -0.91)；P＜0.00001], pleural effusion drainage volume [ MD 822.81, 95%CI(666.46,977.96);P＜0.00001] , FVC%pred [ MD 7.95, 95%CI (4.51,11.40) ; P＜0.00001] , FEV1%pred [ MD 12.67，95%CI (10.09,15.24); P＜0.00001] were significantly different. Continuous variables were represented by mean difference (MD) and the corresponding 95% CI.^36^  Measures of statistical heterogeneity:When P>0.05, it indicated no statistically significant heterogeneity between studies, and a fixed effects model was used for meta-analysis. When P<0.05, heterogeneity between studies was confirmed. Accordingly, the sources of heterogeneity were analyzed. If there was no significant clinical heterogeneity between studies, a random effects model was used for combined analysis, and the results were explained and discussed. After combined analysis, P<0.05 indicated a statistically significant difference.^37^  Combined UK therapy could significantly increase the pleural effusion drainage volume, shorten the absorption time of pleural effusion, reduce pleural thickness, and improve lung function (FEV1% pred, FVC% pred). |  |
|  | 20c | Obvious heterogeneity was observed in the results of these 5 indicators, which may be related to the length of the patient’s disease course, the UK dosage, and the injection method. |  |
|  | 20d | We performed the sensitivity analysis with the method of changing the statistical model. We pooled data using the random-effects model but we also analysed the fixed-efffect model to ensure robustness of the model chosen and susceotibility to outliers. |  |
| Reporting biases | 21 | Funnel plots were drawn with the sample size as the vertical axis and the effect size as the horizontal axis. It was found that the funnel plots for the complete absorption time of pleural effusion (Figure 12), the residual pleural thickness (Figure 13) and the pleural thickness (Figure 14) all appeared to be asymmetric, indicating the presence of publication bias.^37^ As only a small number of studies were included in the subgroup analyses for FVC% pred and FVE1% pred, funnel plot analysis was not conducted. |  |
| Certainty of evidence | 22 | The quality evaluation summary of the 29 included studies are shown respectively in Figure 2. ^4,5, 9-35^ |  |
| **DISCUSSION** | | |  |
| Discussion | 23a | In clinical practice, the intrathoracic injection of hormones and anti-tuberculosis drugs can only reduce inflammatory exudation but not treat the already exuded fluid. An earlier study showed that plasminogen activator inhibitors (PAI) played a decisive role in the fibrinolytic level of pleural effusion, especially PAI-1, which might be related to tissue regeneration, repair and fibrosis development after pleural injury. ^43^ Pollack reported that UK could exert a good therapeutic effect when the formation of pleural fluid had not exceeded 6 weeks and the fibrins had not yet been widely deposited, adhered or separated.44 Huang found that the intrathoracic injection of UK could effectively prevent and treat pleural hypertrophy and adhesion in clinical practice.45 Zhang pointed out that the large amount of fibrin contained in TPE would lead to effusion thickening and generation of protein clots, which might induce the occurrence of multiple pathological processes such as multiloculated and pleural fibrosis.46 In this regard, the plasmin activated by UK can crack the fibrin loculated in the pleural effusion, eliminate the blockage of the fiberloculated to the puncture needle or drainage tube, thus facilitating the drainage of pleural effusion.47 The research by Lin showed that,48 after injection of UK, the pleura was significantly thinned and the cellulose deposition and loculated were significantly reduced compared with the situation after simple conventional anti-tuberculosis treatment. According to the above research results, UK has an obvious effect in the treatment of TPE.  The results of our meta-analysis suggest that the intrathoracic injection of UK is able to promote the absorption of pleural fluid and increase the pleural drainage volume for TPE patients, so as to exert a positive effect in reducing pleural thickness and improving lung function. |  |
|  | 23b | There are still some limitations in this study：① Our meta-analysis only included Chinese and English articles without searching studies in other languages; ② There were differences in terms of the conventional anti-tuberculosis treatment plan, the pleural effusion drainage method, the UK dosage, and the injection method among different studies, so the experimental results were subjected to bias to some extent; ③ The data provided by the included studies were limited, and the course of disease was not investigated; ④ Most of the included studies did not provide a specific description of the double blind methods implemented to the subjects, experimenters, and evaluators, resulting in an increased risk of implementation bias and a generally low Jadad score; ⑤ Most of the included studies had a small sample size, and there might be deviations between the results and the actual situation. |  |
|  | 23c | As only a small number of studies were included in the subgroup analyses for FVC% pred and FVE1% pred, funnel plot analysis was not conducted. |  |
|  | 23d | Our meta-analysis of 29 RCTs showed that the UK treatment group had a significant increase in the pleural effusion drainage volume and lung function (FEV1% pred, FVC% pred), and a significant decrease in the pleural thickness and absorption time of pleural effusion. All these differences were statistically significant (P<0.05), suggesting that the combined UK therapy could significantly increase the pleural effusion drainage volume, shorten the absorption time of pleural effusion, reduce pleural thickness, and improve lung function (FEV1% pred, FVC% pred). Our study can provide a useful reference for clinical practice. But given the limitations of this study, our findings need to be further verified by more high-quality, large-scale clinical studies both domestically and internationally. |  |
| **OTHER INFORMATION** | | |  |
| Registration and protocol | 24a | Provide registration information for the review, including register name and registration number, or state that the review was not registered. |  |
|  | 24b | Indicate where the review protocol can be accessed, or state that a protocol was not prepared. |  |
|  | 24c | Describe and explain any amendments to information provided at registration or in the protocol. |  |
| Support | 25 | Describe sources of financial or non-financial support for the review, and the role of the funders or sponsors in the review. |  |
| Competing interests | 26 | Declare any competing interests of review authors. |  |
| Availability of data, code and other materials | 27 | Report which of the following are publicly available and where they can be found: template data collection forms; data extracted from included studies; data used for all analyses; analytic code; any other materials used in the review. |  |

*From:*  Page MJ, McKenzie JE, Bossuyt PM, Boutron I, Hoffmann TC, Mulrow CD, et al. The PRISMA 2020 statement: an updated guideline for reporting systematic reviews. BMJ 2021;372:n71. doi: 10.1136/bmj.n71

For more information, visit: <http://www.prisma-statement.org/>
