# Supplementary material for: Urokinase in the treatment of tuberculous pleurisy: a systematic review and meta-analysis
Source: BMC Infect Dis. 2024 Feb 24;24:258. doi: 10.1186/s12879-024-08975-0 (PMC10893646; doi:10.1186/s12879-024-08975-0)
Supplement: Supplementary file 2 — Additional file 2. [file 12879_2024_8975_MOESM2_ESM.docx]

**Chinese Database**

**1、CBM Retrieve time：2023-2-11**

1) "Tuberculosis, Pleural"[unweighted:extend]

2) pleural tuberculosis

3) Tuberculous pleurisy

4) Tuberculosis, Pleural

5) Tuberculous pleural effusion

6) tuberculous pleural effusion

7) tuberculous pleural effusion

8) (#7) OR (#6) OR (#5) OR (#4) OR (#3) OR (#2) OR (#1)

9) "Urokinase type plasminogen activato"[unweighted:extend]

10) U plasma proenzyme agonist

11) U-PA

12) urine plasminogen activator

13) urokinase

14) Yakinase

15) Renal plasminogen activator

16) single chain urokinase plasminogen activator

17) Renokinase

18) Urokinase-Type Plasminogen Activator

19) (#18) OR (#17) OR (#16) OR (#15) OR (#14) OR (#13) OR (#12) OR (#11) OR (#10) OR (#9)

20) "RCT"[unweighted:extend]

21) Randomized Controlled Trial

22) random

23) Controlled Clinical Trials

24) RCT

25) (#24) OR (#23) OR (#22) OR (#21) OR (#20)

26) ((#25) AND (#19) AND (#8)) AND 2000-2023[time]

**2、CNKI Retrieve time：2023-2-11**

(theme:Tuberculosis, Pleural(accurate) ) OR (theme: Tuberculosis, Pleural (accurate) ) OR (theme:pleural tuberculosis(accurate) ) OR (theme:Tuberculous pleurisy(accurate) ) OR (theme: Tuberculous pleural effusion(accurate) ) OR (theme:tuberculous pleural effusion(accurate) ) OR (theme: tuberculous pleural effusion (accurate) ) AND ( (theme: Urokinase type plasminogen activator(accurate)) OR (theme: Urokinase-Type Plasminogen Activator (accurate) )OR (theme: U plasma proenzyme agonist(accurate) ) OR (theme: U-PA (accurate) ) OR (theme:urine plasminogen activator(accurate) ) OR (theme:urokinase (accurate) ) OR (theme:Yakinase(accurate) ) OR (theme:Renal plasminogen activator (accurate) ) OR (theme:Single chain Urokinase type plasminogen activator(accurate) ) OR (theme: Renokinase (accurate) ) ) AND( (abstract:RCT(accurate) ) OR (abstract: Randomized Controlled Tria| (accurate) ) OR (abstract:Random(accurate) ) OR (abstract:Controlled Clinical Trials(accurate) )

OR (abstract: RCT (accurate) ) )

**3、Wanfang Database Retrieve time：2023-2-11**

(theme:(Tuberculous pleura or Tuberculosis, Pleural or pleural tuberculosis or Tuberculous pleurisy or Tuberculous pleural effusion or tuberculous pleural effusion or tuberculous pleural effusion) and theme:(Urokinase type plasminogen activatoror Urokinase- Type Plasminogen Activator or U plasma proenzyme agonist or U-PA or urine plasminogen activator or urokinase or Yakinase or Renal plasminogen activator or Single chain Urokinase type plasminogen activator or Renokinase) and theme:(RCT or Randomized Controlled Trial or Random or Controlled Clinical Trials or RCT)) and Date:2000-2023

**English Database**

**4、PubMed Retrieve time 2023-2-6**

(((((((((Pleural Tuberculoses[Title/Abstract]) OR (Pleural Tuberculosis[Title/Abstract])) OR (Tuberculoses, Pleural[Title/Abstract])) OR (Pleurisy, Tuberculous[Title/Abstract])) OR (Pleurisies, Tuberculous[Title/Abstract])) OR (Tuberculous Pleurisies[Title/Abstract])) OR (Tuberculous Pleurisy[Title/Abstract])) OR ("Tuberculosis, Pleural"[Mesh])) AND (("Urokinase-Type Plasminogen Activator"[Mesh]) OR ((((((((((((Urokinase Type Plasminogen Activator[Title/Abstract]) OR (Plasminogen Activator, Urokinase-Type[Title/Abstract])) OR (U-Plasminogen Activator[Title/Abstract])) OR (U Plasminogen Activator[Title/Abstract])) OR (U-PA[Title/Abstract])) OR (Urinary Plasminogen Activator[Title/Abstract])) OR (Urokinase[Title/Abstract])) OR (Renokinase[Title/Abstract])) OR (Abbokinase[Title/Abstract])) OR (Kidney Plasminogen Activator[Title/Abstract])) OR (Single-Chain Urokinase-Type Plasminogen Activator[Title/Abstract])) OR (Single Chain Urokinase Type Plasminogen Activator[Title/Abstract])))) AND (randomized controlled trial[Publication Type] OR randomized[Title/Abstract] OR placebo[Title/Abstract])

**5、Embase Retrieve time：2023-2-16**

#40. #12 AND #31 AND #38 AND [2000-2023]/py

#39. #12 AND #31 AND #38

#38. #32 OR #33 OR #34 OR #35 OR #36 OR #37

#37. 'trial, randomized controlled':ab,ti

#36. 'randomized controlled study':ab,ti

#35. 'randomised controlled trial':ab,ti

#34. 'randomised controlled study':ab,ti

#33. 'controlled trial, randomized':ab,ti

#32. 'randomized controlled trial'/exp OR 'randomized

controlled trial'

#31. #13 OR #14 OR #15 OR #16 OR #17 OR #18 OR #19 OR

#20 OR #21 OR #22 OR #23 OR #24 OR #25 OR #26 OR

#27 OR #28 OR #29 OR #30

#30. 'urokine':ab,ti

#29. 'urokinase-type plasminogen activator':ab,ti

#28. 'urokinase type plasminogen activator':ab,ti

#27. 'urokinase plasminogen activator':ab,ti

#26. 'urinary type plasminogen activator':ab,ti

#25. 'urinary plasminogen activator':ab,ti

#24. 'ukidan':ab,ti

#23. 'two chain urokinase type plasminogen

activator':ab,ti

#22. 'rheotromb':ab,ti

#21. 'pro-urokinase':ab,ti

#20. 'plasminogen activator, urinary':ab,ti

#19. 'medacinase':ab,ti

#18. 'kinlytic':ab,ti

#17. 'corase':ab,ti

#16. 'alphakinase':ab,ti

#15. 'actosolv':ab,ti

#14. 'abbokinase open-cath':ab,ti

#13. 'urokinase'/exp OR 'urokinase'

#12. #1 OR #2 OR #3 OR #4 OR #5 OR #6 OR #7 OR #8 OR

#9 OR #10 OR #11

#11. 'tuberculous pleurisy'/exp OR 'tuberculous

pleurisy'

#10. 'tuberculous pleuritis':ab,ti

#9. 'tuberculous pleural effusion':ab,ti

#8. 'tuberculosis, pleural':ab,ti

#7. 'tuberculosis pleurae':ab,ti

#6. 'tuberculosis of the pleura':ab,ti

#5. 'pleuritis tuberculosa':ab,ti

#4. 'pleurisy, tuberculous':ab,ti

#3. 'pleural tuberculosis':ab,ti

#2. 'pleural tb':ab,ti

#1. 'pleura tuberculosis':ab,ti

**6、Cochrane Library Retrieve time：2023-2-11**

#1   MeSH descriptor: [Tuberculosis,Pleural] explode all trees
#2   (Pleural Tuberculosis or Pleural Tuberculoses or Tuberculoses, Pleural or Tuberculous Pleurisy or Tuberculous Pleurisies or Pleurisies, Tuberculous or Pleurisy, Tuberculous):ti,ab,kw
#3   (tuberculous pleural effusion):ti,ab,kw
#4   #1 or #2 or #3
#5   MeSH descriptor: [Urokinase-Type Plasminogen Activator] explode all trees
#6   (Abbokinase or Single Chain Urokinase Type Plasminogen Activator or Single-Chain Urokinase-Type Plasminogen Activator or Kidney Plasminogen Activator or Renokinase or Plasminogen Activator, Urokinase-Type or U-PA or U Plasminogen Activator or Urokinase Type Plasminogen Activator or Urinary Plasminogen Activator or Urokinase or U-Plasminogen Activator):ti,ab,kw
#7   #5 or #6
#8   (Randomized Controlled Trial):ti,ab,kw
#9   #4 or #7 or #8

**Limit time range**2000-2023

**7、Web of science Retrieve time：2023-2-11**

[((TS=(Tuberculosis, Pleural OR Pleural Tuberculoses OR Pleural Tuberculosis OR Tuberculoses, Pleural OR Pleurisy, Tuberculous OR pleuresies, Tuberculous OR Tuberculous pleuresies OR Tuberculous Pleurisy)) AND TS=(Urokinase-Type Plasminogen Activator OR Urokinase Type Plasminogen Activator OR Plasminogen Activator, Urokinase-Type OR U-Plasminogen Activator OR U Plasminogen Activator OR U-PA OR Urinary Plasminogen Activator OR Urokinase OR ribokinase OR autokinase OR Kidney Plasminogen Activator OR Single-Chain Urokinase-Type Plasminogen Activator OR Single Chain Urokinase Type Plasminogen Activator)) AND TS=(randomized controlled trial)](https://www.webofscience.com/wos/woscc/summary/381b962f-8d8e-4fcc-a08b-d3fbd43a150f-7113fbba/relevance/1)
